# Jail fever (epidemic typhus) outbreak in Burundi.

**DOI:** 10.3201/eid0303.970313

**Published:** 1997

**Authors:** D Raoult, V Roux, J B Ndihokubwayo, G Bise, D Baudon, G Marte, R Birtles

**Affiliations:** Université de la Méditerranée, Faculté de Médecine, Marsaille, France.

## Abstract

We recently investigated a suspected outbreak of epidemic typhus in a jail in Burundi. We tested sera of nine patients by microimmunofluorescence for antibodies to Rickettsia prowazekii and Rickettsia typhi. We also amplified and sequenced from lice gene portions specific for two R. prowazekii proteins: the gene encoding for citrate synthase and the gene encoding for the rickettsial outer membrane protein. All patients exhibited antibodies specific for R. prowazekii. Specific gene sequences were amplified in two lice from one patient. The patients had typical clinical manifestations, and two died. Molecular techniques provided a convenient and reliable means of examining lice and confirming this outbreak. The jail-associated outbreak predates an extensive ongoing outbreak of louse-borne typhus in central eastern Africa after civil war and in refugee camps in Rwanda, Burundi (1), and Zaire.


### Jail Fever (Epidemic Typhus) Outbreak in Burundi

We recently investigated a suspected outbreak of epidemic typhus in a jail in Burundi. We tested sera of nine patients by microimmunofluorescence for antibodies to *Rickettsia prowazekii* and *Rickettsia typhi*. We also amplified and sequenced from lice gene portions specific for two *R. prowazekii* proteins: the gene encoding for citrate synthase and the gene encoding for the rickettsial outer membrane protein. All patients exhibited antibodies specific for *R. prowazekii*. Specific gene sequences were amplified in two lice from one patient. The patients had typical clinical manifestations, and two died. Molecular techniques provided a convenient and reliable means of examining lice and confirming this outbreak. The jail-associated outbreak predates an extensive ongoing outbreak of louse-borne typhus in central eastern Africa after civil war and in refugee camps in Rwanda, Burundi (1), and Zaire.

Typhus group rickettsioses are distributed worldwide. Rickettsial typhus has two forms: endemic (murine) typhus and epidemic typhus. Endemic typhus is caused by *R. typhi*, usually transmitted to humans by the rat flea, *Xenopsylla cheopis*; epidemic typhus is caused by *R. prowazekii*, transmitted by the body louse, *Pediculus humanus humanus* (2). In the United States, *R. prowazekii* has also been found in a sylvatic cycle involving flying squirrels and their ectoparasites (3). Typhus group rickettsioses, especially epidemic typhus, are associated with wars and human disasters.

During World Wars I and II, typhus spread through North Africa, the Pacific Islands, and Europe, especially in German concentration camps. In North Africa, the epidemics involved mainly civilian populations. U.S. forces were protected by the first vaccine applied on a large scale, the Cox-type vaccine (4). Epidemic typhus began to decline at the end of World War II when DDT started to be used as an insecticide. Ethiopia, Rwanda, Nigeria, and Burundi were the main foci of louse-borne typhus in Africa in the 1970s. Ethiopia reported 1,931 cases in 1983 and 4,076 cases in 1984 (5), with a death rate of 3.8%(6). However, these data were difficult to interpret because serologic testing was usually performed with inadequate techniques, and epidemiologic data were unavailable.

Political instability in Eastern European and African countries has recently caused conflicts in regions where the risk for typhus outbreaks is very high. In Yugoslavia and Rwanda, older persons who contracted epidemic typhus as youths can develop recrudescent illness and become the source of resurgent epidemics if louse infestation becomes prevalent. Recently, an outbreak of typhus was suspected in a Burundi jail; we diagnosed epidemic typhus by amplifying and sequencing portions of the citrate synthase and the rOmpB genes of *R. prowazekii* in lice.

### Patients

In a jail in N'Gozi, Burundi, a local nurse observed patients with abrupt fevers of up to 40°C in association with a body louse outbreak from December 1995 to January 1996. Typhus was suspected, and chloramphenicol was given orally or intravenously. Blood specimens were obtained from nine patients (all young black men), and sera were sent to Marseille for serologic testing. Two lice per patient were obtained from the clothes of Patients Four, Five, Seven, Eight, and Nine; clinical information was also obtained (Table).

### Serologic Investigation

Patients' sera were tested by microimmunofluorescence. Vero cell–grown rickettsia were purified (7) and placed on slides as spots. *R. prowazekii* Breinl strain and *R. typhi* Wilmington strain were used. Both antigens were placed on either side of the same slide to compare the serum reactivity with the two antigens. Sera were diluted from 1/32 to 1/4,096. Immunoglobulin G (IgG) antibodies were detected by gamma chain specific anti-human Ig (Bio Merieux, Marcy l'Etoile, France), and IgM was detected after IgG was removed by RF adsorbent (Behring) (7). The diagnostic cutoff for microimmunofluorescence in

Table. Clinical and serologic data (by microimmunofluorescence) for nine patients with louse-borne typhus

| Patient number | One | Two | Three | Four | Five | Six | Seven | Eight | Nine |
|---|---|---|---|---|---|---|---|---|---|
| Age (yrs) | 28 | 21 | 33 | 20 | 27 | 25 | 28 | 50 | 27 |
| Onset date | 1/25/96 | 1/26/96 | 1/6/96 | 1/19/96 | 1/20/96 | 1/19/96 | 12/16/95 | 1/13/96 | 1/20/96 |
| Peak fever (°C) | 40 | 39.7 | 40 | 39.5 | 40 | 39.5 | 39.7 | 39 | 40 |
| Neurologic signs | no | no | yes | yes | yes | yes | yes | yes | yes |
| mental confusion | - | - | yes | no | yes | yes | yes | yes | yes |
| seizures | - | - | no | yes | no | no | no | no | no |
| coma | - | - | yes | no | no | no | yes | yes | no |
| Rash | yes | no | yes | no | no | no | no | no | no |
| purpuric | no | - | yes | - | - | - | - | - | - |
| Pulmonary signs | yes | no | no | yes | yes | yes | yes | no | no |
| cough | yes | - | - | yes | yes | no | no | - | - |
| dyspnea | yes | - | - | no | no | yes | yes | - | - |
| Vomiting | yes | no | yes | yes | no | yes | yes | no | no |
| Splenomegaly | no | yes | yes | no | no | no | yes | no | no |
| Hypotension | yes | no | no | no | yes | no | yes | no | yes |
| Death | no | no | no | no | no | no | yes | no | yes |
| Anti-*R. prowazekii* IgG titer | 256 | 1024 | 2048 | 256 | 256 | 512 | 4096 | 1024 | 256 |
| Anti-*R. prowazekii* IgM titer | 256 | 512 | 512 | 256 | 512 | 512 | 256 | 1024 | 512 |
| Anti-*R. typhi* IgG titer | 64 | 512 | 128 | 256 | 64 | 0 | 1024 | 1024 | 64 |
| Anti-*R. typhi* IgM titer | 256 | 512 | 512 | 256 | 512 | 512 | 256 | 1024 | 512 |

diagnosing rickettsiosis is 1/128 for IgG and 1/64 for IgM (7,8). All nine patients were positive for both antigens; however, titers were identical for both antigens in Patients Four and Eight. In the other patients, antibodies were higher for *R. prowazekii* than for *R. typhi* by one-to-five dilution for IgG. IgM antibodies were at the same level against both antigens in all patients (Table).

### Polymerase Chain Reaction (PCR) Detection in Lice

Lice were sent to Marseille and arrived dry; they were crushed, and DNA suitable for use as a template in PCR was extracted using QIAGEN columns (QIAamp Tissue Kit, QIAGEN, Hilden, Germany). Citrate synthase PCR amplification incorporated primers 120-M59 (CCGCAGGGTTGGTAACTGC) (9) and 120-508 (CCAAGTGATAAGAGGAGCTT) selected from the *R. prowazekii ompB* sequence (10). The citrate synthase amplicons were subjected to restriction fragment length polymorphism analysis using the enzyme *AluI* and were identical to that previously described for *R. prowazekii* (11). Sequence determinations were performed by using an automated laser fluorescent DNA sequencer as previously described (12). The citrate synthase and rOmpB sequences obtained from the lice had 100% homology with that of *R. prowazekii* strain Breinl. Of the 10 tested, two lice from the same patient (four) were positive.

### Cause, Symptoms, and Diagnosis

Social conditions are critical factors influencing the reemergence of disease. Louse infestation occurs when cold weather, poor hygiene, and poverty are prevalent; these conditions have been present in refugee camps in Rwanda and Zaire recently (13,14). Louse infestation has also been found in several eastern European countries and in homeless persons in western Europe (15).

Epidemic typhus was suspected in a refugee camp in Goma, Zaire, in 1994, but the outbreak was unconfirmed (14). Typhus is usually exanthematic, and a careful clinical examination will find a rash in more than half of the cases. In the present series, clinical data were recorded retrospectively mainly on the basis of a nurse's report and are probably incomplete (Table). In an outbreak, looking for exanthema is critical for typhus suspicion. The typhus exanthema is frequently purpuric and is observed even on dark skin in 33% of cases (6). The other typical symptoms are neurologic signs including seizures, coma, and mental confusion, which gave the disease its name (ORIG: from the Greek word "typhos," which means smoke, cloud, stupor arising from fever). Neurologic involvement was noted in seven of the nine cases: symptoms included coma, seizures, mental confusion, and delirium. Rash was described in only two patients (Figure); splenomegaly in three patients; cough in three patients; dyspnea in three patients; and hypotension in four patients. Two patients died. The other patients recovered rapidly after receiving chloramphenicol. The prevalence of pulmonary symptoms is high in general as noticed here.

In situations like those observed in this jail, one can suspect either murine or epidemic typhus. Murine typhus is less severe. However, in poor hygienic conditions, rats are prevalent, and the risk exists for both diseases. Consequently, in such situations it is critical to discriminate between *R. prowazekii* and *R. typhi* infections because they do not have the same epidemic potential. Diagnosis is usually provided by micro-immunofluorescence; in some cases antibody levels are sufficient to differentiate between the two diseases. IgG titers are usually different, but IgM titers are not. When, as in Patients Four and Eight, antibodies are at the same level, cross-adsorption, a tedious and expensive procedure requiring large amounts of antigens and serum (not available in our study [16]), can differentiate between the two diseases. The use of arthropod vectors for gene amplification and disease recognition has been reported (17). In a case of laboratory-acquired human typhus, diagnosis was confirmed by PCR (18). However, in the present work, we report for the first time the use of the louse in diagnosing epidemic typhus in the field.

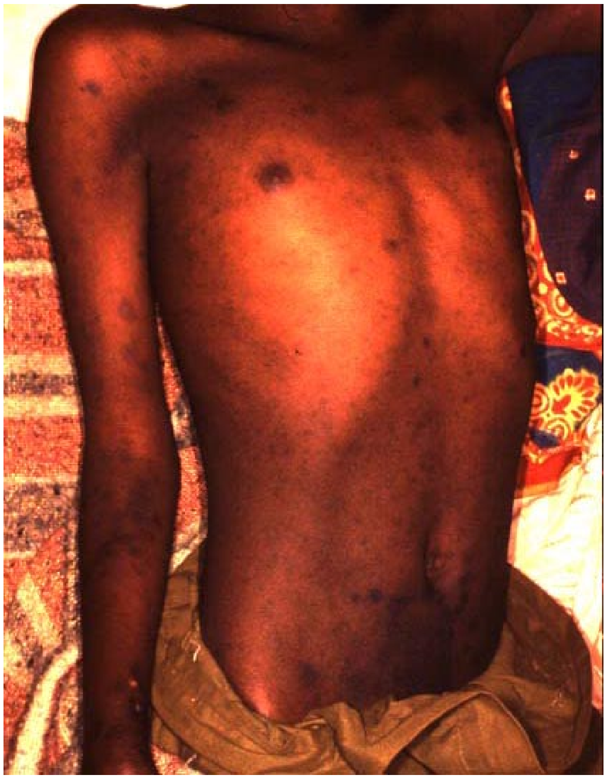

Figure. Rash in a man with epidemic typhus in Burundi.

The confirmation of a typhus outbreak in Burundi is of concern, given the relative political instability in this area of the world. A preliminary report from the World Health Organization discusses a current large outbreak in Burundi with an estimated 24,000 cases (1). It is uncertain if the outbreak we observed is related to the current epidemic, but our present work confirms continuing cycles of louse-borne typhus in Burundi. Delousing (14) is critical to typhus prevention. The treatment for typhus is simple and inexpensive: a single dose of 200 mg of doxycycline provides cure (19).

We stress the major risk for exanthematic typhus in central eastern Africa including Burundi, Zaire, and Rwanda and the importance of sending lice to reference laboratories to identify the pathogens when a typhus outbreak is observed. Molecular identification by PCR and sequencing offers a rapid, sensitive, and specific identification method for *Rickettsia,* even when epidemiologic investigations are undertaken.

**D. Raoult,* V. Roux,* J.B. Ndihokubwayo,† G. Bise,‡ D. Baudon,§ G. Martet,§ and R. Birtles***
*Université de la Méditerranée, Faculté de Médecine, Marseille, France; †CHU de Bujumbura, Laboratoire de Biologie Médicale, République du Burundi; ‡World Health Organization, CICR; Geneva, Switzerland; §Institut de Médecine Tropicale, Service de Santé des Armées, Marseille, France

### References

1. World Health Organization. A large outbreak of epidemic louse-borne typhus in Burundi. Wkly Epidemiol Rec 1997;21:152-3.
2. Azad AF. Relationship of vector biology and epidemiology of louse- and flea-borne rickettsioses. In: Walker DH, editor. Biology of rickettsial diseases. Boca Raton (FL): CRC Press; 1991. p. 51-61.
3. McDade JE. Evidence of *Rickettsia prowazekii* infections in the United States. Am J Trop Med Hyg 1980;29:277-83.
4. Woodward TE. Rickettsial vaccines with emphasis on epidemic typhus. Initial report of an old vaccine trial. S Afr Med J 1986; Suppl:73-6.

5. World Health Organization. Louse-borne typhus, 1983-1984. Wkly Epidemiol Rec 1986;7:49-50.
6. Perine PL, Chandler BP, Krause DK, McCardle P, Awoke S, Habte-Gabr E, et al. A clinico-epidemiological study of epidemic typhus in Africa. Clin Infect Dis 1992;14:1149-58.
7. Eremeeva ME, Balayeva NM, Raoult D. Serological response of patients suffering from primary and recrudescent typhus: comparison of complement fixation reaction, Weil-Felix test, microimmunofluorescence, and immunoblotting. Clin Diag Lab Immunol 1994;1:318-24.
8. Ormsbee R, Peacock M, Philip R, Casper E, Plorde J, Gabre-Kidan T, Wright L. Serologic diagnosis of epidemic typhus fever. Am J Epidemiol 1977;105:261-71.
9. Wood DO, Williamson LR, Winkler HH, Krause DC. Nucleotide sequence of the *Rickettsia prowazekii* citrate synthase gene. J Bacteriol 1987;169:3564-72.
10. Regnery RL, Spruill CL, Plikaytis BD. Genomic identification of rickettsiae and estimation of intraspecies sequence divergence for portions of two rickettsial genes. J Bacteriol 1991;173:1576-89.
11. Carl M, Dobson ME, Ching WM, Dasch GA. Characterization of the gene encoding the protective paracrystalline-surface-layer protein of *Rickettsia prowazekii:* presence of a truncated identical homolog in *Rickettsia typhi*. Proc Natl Acad Sci U S A 1990;87:8237-41.
12. Joblet C, Roux C, Drancourt M, Gouvernet J, Raoult D. Identification of *Bartonella* (*Rochalimaea*) species among fastidious Gram-negative bacteria based on the partial sequence of the citrate-synthase gene. J Clin Microbiol 1995;33:1879-83.
13. World Health Organization. Global surveillance of rickettsial diseases: memorandum from a WHO meeting. Bull World Health Organ 1993;71:293-6.
14. World Health Organization. Epidemic typhus risk in Rwandan refugee camps. Wkly Epidemiol Rec 1994;34:259
15. Stein A, Raoult D. Return of trench fever. Lancet 1995;345:450-1.
16. Raoult D, Dasch GA. Immunoblot cross-reactions among *Rickettsia, Proteus* spp. and *Legionella* spp. in patients with Mediterranean spotted fever. FEMS Immunol Med Microbiol 1995;11:13-8.
17. Azad AF, Sacci JB, Nelson WM, Dasch GA, Schmidtmann ET, Carl M. Genetic characterization and transovarial transmission of a typhus-like rickettsia found in cat fleas. Proc Natl Acad Sci U S A 1992;89:43-6.
18. Carl M, Tibbs CW, Dobson ME, Paparello SF, Dasch GA. Diagnosis of acute typhus infection using the polymerase chain reaction. Ann N Y Acad Sci 1990;590:439-44.
19. Perine PL, Krause DW, Awoke S, McDade JE. Single-dose doxycycline treatment of louse-borne relapsing fever and epidemic typhus. Lancet 1974;2:742-4.
